# FMOD Alleviates Depression-Like Behaviors by Targeting the PI3K/AKT/mTOR Signaling After Traumatic Brain Injury

**DOI:** 10.1007/s12017-024-08793-2

**Published:** 2024-06-12

**Authors:** Xuekang Huang, Ziyu Zhu, Mengran Du, Chenrui Wu, Jiayuanyuan Fu, Jie Zhang, Weilin Tan, Biying Wu, Lian Liu, Z. B. Liao

**Affiliations:** Department of Neurosurgery, The First Affiliated Hospital of Chongqing Medical University, No. 1 Youyi Road, Yuanjiagang, Yuzhong District, Chongqing, 400016 China

**Keywords:** TBI, Depression, FMOD, Synaptic plasticity, PI*3K/AKT/mTOR*

## Abstract

Depression frequently occurs following traumatic brain injury (TBI). However, the role of Fibromodulin (FMOD) in TBI-related depression is not yet clear. Previous studies have suggested FMOD as a potential key factor in TBI, yet its association with depression post-TBI and underlying mechanisms are not well understood. Serum levels of FMOD were measured in patients with traumatic brain injury using qPCR. The severity of depression was assessed using the self-depression scale (SDS). Neurological function, depressive state, and cognitive function in mice were assessed using the modified Neurological Severity Score (mNSS), forced swimming test (FST), tail suspension test (TST), Sucrose Preference Test (SPT), and morris water maze (MWM). The morphological features of mouse hippocampal synapses and neuronal dendritic spines were revealed through immunofluorescence, transmission electron microscopy, and Golgi-Cox staining. The protein expression levels of FMOD, MAP2, SYP, and PSD95, as well as the phosphorylation levels of the PI3K/AKT/mTOR signaling pathway, were detected through Western blotting. FMOD levels were decreased in TBI patients’ serum. Overexpression of FMOD preserved neuronal function and alleviated depression-like behaviour, increased synaptic protein expression, and induced ultrastructural changes in hippocampal neurons. The increased phosphorylation of PI3K, AKT, and mTOR suggested the involvement of the PI3K/AKT/mTOR signaling pathway in FMOD’s protective effects. FMOD exhibits potential as a therapeutic target for depression related to TBI, with its protective effects potentially mediated through the PI3K/AKT/mTOR signaling pathway.

**Supplementary Information:**

The online version contains supplementary material available at 10.1007/s12017-024-08793-2.

## Introduction

Traumatic brain injury (TBI) is a critical global health problem with devastating lifelong consequences, it not only leads to brain dysfunction but also psychiatric disorders (Pavlovic et al., 2019). Depression is one of the most common psychiatric sequelae ensuing from TBI, it might influence 25–50% of TBI patients (Jahan & Tanev, 2023). Studies suggested that TBI-related depression significantly affected personal life quality and even resulted in lifelong impairment (Boyko et al., 2023). Nevertheless, much remains unclear about the complicated neurobiological alternations of post-TBI depression.

Fibromodulin (FMOD) is a secreted protein and a member of the Small leucine-rich proteoglycans family, which is primarily expressed in the extracellular matrix (ECM) (Jan et al., 2016). ECM remodeling is known to significantly impact TBI pathophysiology (George & Geller, 2018). A previous study reported FMOD as a key regulatory gene participating in the ECM-related regulation after TBI, potentially contributing to tissue repair and remodeling post-TBI (Meng et al., 2017). Yet, the biological function of FMOD in depression after TBI remains unclear. Interestingly, decreased expression of FMOD in the brain has been reported in depression after Chronic Immobilization Stress (Jung et al., 2012). Alterations in FMOD expression in the brain suggested its potential correlation with depression. It is worth noting that the synaptic inhibition in the Medial Prefrontal Cortex and hippocampus could influence the occurrence of depression (Abdallah et al., 2015). Therefore, we hypothesized that the FMOD, which exists in ECM, could ameliorate post-TBI depression by modulating synaptic plasticity.

Numerous signaling pathways are involved in the pathophysiology of TBI. One of these pathways, the PI3K/AKT/mTOR pathway, is known to play a crucial role (Mezhlumyan et al., 2022; Raab-Graham et al., 2006). Activating the PI3K/AKT/mTOR signaling could suppress neural inflammation, autophagy, and apoptosis, then increase synaptic protein synthesis after TBI (Ding et al., 2023; Jan et al., 2016). Furthermore, this pathway is also involved in the resolving of depression symptoms by regulating the function of the synapses and promoting synaptogenesis (Dwyer & Duman, 2013; Fakhri et al., 2021). PI3K/Akt/mTOR signaling cascade might be the vital transduction in TBI-related depression. But now, scarce studies have explored whether the FMOD acted on PI3K/AKT/mTOR signaling and linked this probable mechanism to depression after TBI.

Herein, this study was conducted to reveal the effects of FMOD in post-TBI depression and its potential mechanism. The self-rating depression scales (SDS) were used to determine the depression of TBI patients, the reduction of FMOD was found and was associated with TBI-related depression. We next investigated the efficacy of FMOD in the experiments in mice and primary neuronal cells. At last, the favorable outcomes of overexpressing FMOD in depression after TBI were exerted through the PI3K/Akt/mTOR signaling pathway.

## Materials and Methods

### Blood Specimen Collection

Venous blood was collected from 40 TBI patients upon admission, with an additional 20 healthy volunteers serving as controls. Both groups of patients were randomly selected based on age, gender, and BMI. (P > 0.05). Patient selection criteria were: a Glasgow Coma Scale score of 8–13; age between 18 and 55 years; TBI within the day before admission. Exclusion criteria were severe complications such as significant infections upon admission; a history of intracranial lesions, psychiatric disorders, or similar injuries; pregnancy or lactation. Detailed patient information is provided in Supplementary Table 1.

### Depression-Like Behavior Tests in Patients

We utilized the Chinese version of the SDS to assess depressive symptoms (Zung, 1965). Developed by Zung in 1965, the SDS consists of 20 items covering emotional, psychological, and cognitive aspects. Elevated scores signify more pronounced depressive symptoms. For this study, we established a threshold of 53, deeming total scores exceeding this value indicative of the presence of depressive symptoms (Xing et al., 2021).

### Animals Experiments

The sample size for animal specimens was calculated using an online tool (http://www.lasec.cuhk.edu.hk/sample-size-calculation.html). A cohort of 105 male C57BL/6 mice, aged 6–8 weeks, were procured from the Animal Experimental Center of Chongqing Medical University and received appropriate care in accordance with the guidelines set by the Institutional Animal Care and Use Committee of Chongqing Medical University. The mice were allocated to different groups, including TBI, TBI + FMOD, TBI + sh-FMOD, TBI + sh-NC, and Sham, using a simple randomization approach. Fifteen mice from each group were involved in the behavioral experiments and subsequently used for sample collection and analysis at the conclusion of the study. Additionally, an extra 30 mice (five per group) were utilized to determine the time course expression levels of FMOD. Throughout the entire experimental and data analysis processes, the researchers remained unaware of the animals’ grouping.

### TBI Mouse Model Establishment

To mimic TBI in mice, controlled cortical impact (CCI) was selected, based on previous studies (Osier & Dixon, 2016; Wu et al., 2022). In summary, mice were initially anesthetized with 3% isoflurane. Anesthesia was then maintained at a level of 1.5% isoflurane, and the mice were subsequently secured in a stereotactic frame. A bone flap above the left frontal lobe was removed, with surgical coordinates centered on the posterior fontanelle (2 mm posterior, 2 mm lateral). CCI was performed using an electronically controlled pneumatic impact device (PSI, USA). A 3 mm-diameter flat impactor, penetrating to a depth of 1.5 mm at a velocity of 5 m/s, was applied to the surgical site on the stereotaxic frame, with a dwell time of 100 ms. In the sham group, mice were anaesthetised solely for the scalp incision and craniotomy, and were subsequently transferred back to the recovery cage after resuscitation. In the remaining group, mice had their scalps sutured immediately after impact and were placed on a heating pad maintained at a constant temperature of 37 °C. Following resuscitation, these mice were also transferred back to the recovery cage.

### Adeno-Associated Virus (AAV) Intracerebroventricular Injection

On day 7 after TBI, mice were anesthetized in the same manner as the CCI procedure and were mounted on a stereotactic device (Stoelting, USA). Injections were made to the left lateral ventricle (coordinates from the bregma: anteroposterior (AP) − 0.5 mm, lateral (L) − 1 mm, dorsoventral (DV) − 3 mm). The AAV-containing solution was loaded into a sterilized 5 µl Hamilton syringe (Hamilton, USA). Animals received 2 µl of either FMOD short hairpin (sh) RNA (sh-FMOD) or empty vector (sh-NC) (Qingke Biotech, Beijing, China) at a rate of 0.4 µl/min (exact sequence can be found in Supplementary Table 2); the needle was left in place for another 5 min before being slowly retracted after injection.

### Intranasal Delivery of FMOD

The acclimation training started on day 9 post-TBI and lasted for seven days, during which all groups were administrated with 0.9% saline (20 µl/mice/day). On day16 after TBI, the TBI + FMOD group received a 5 µg of FMOD (Sino Biological, 11514-H02H, China) formulated in 20 µl phosphate buffered saline once a day, while the remaining groups received the same amount of saline; the solutions were given intranasally for five days.

### Modified Neurological Severity Score (mNSS) Test

On day 28 after TBI, we evaluated the neurological recovery of rats using the mNSS test, which assesses various aspects, including motor coordination, sensory function, balance, and reaction time, providing a comprehensive evaluation of rodent neurological function. Scores on the mNSS range from 0 to 18, and a higher total score reflects more severe neurological deficits.

### Forced Swimming Test (FST)

The FST is a widely used and validated assessment for depression in rodents, reflecting their behavioural responses to depressive or antidepressant compounds (Cryan et al., 2002). During the test, mice respond to unavoidable acute stressors by oscillating between struggling and remaining immobile. The test involves individually housing mice in transparent cylinders (25 cm high and 20 cm in diameter) filled with water (24 °C) at a depth of 19 cm. The total stress exposure duration is 6 min, and the quiescence duration represents the period in which a lack of avoidance behavior is observed during the last 4 min of the experiment (Ghasemi et al., 2009).

### The Tail Suspension Test (TST)

The TST is a widely employed experimental approach to evaluate antidepressant effects in mice or rats (Zhang et al., 2019). Following acclimation to a tranquil and dim environment, mice are individually suspended by their tails on a horizontal bar for 6 min. Different behaviors, including struggling, curling, and immobility, are observed throughout this period, with a specific focus on immobility during the last 4 min.

### The Sucrose Preference Test (SPT)

The SPT is utilized for the evaluation of hedonic-lack behavior, a fundamental manifestation of depression. The SPT methodology, with minor adjustments derived from prior investigations (Ghasemi et al., 2009), included acclimating animals to 1% sucrose solution (w/v) and water for 24 h, succeeded by a 12-h period of water and food deprivation. Following this, mice were exposed to two bottles, each containing 150 ml of 1% sucrose solution (w/v) or water. The weights of the bottles were measured both before and after the testing. The sucrose preference results were calculated as the ratio of consumed sucrose solution to the overall liquid intake.

### Electroencephalogram (EEG)

On day 28 following TBI, we conducted EEG to assess sleep patterns and cerebral electrical activity in mice. Sleep was induced by administering 3% chloral hydrate (5 ml/kg, Macklin, Cat#C804539, China). Active electrodes were placed under the scalp on both sides of the parietal cortex, and reference electrodes were situated above the mastoid. Cerebral electrical activity was monitored using an EEG device (Natus Neurology Inc., Middleton, Wisconsin, USA), allowing the visualization of sleep electroencephalogram and facilitating the analysis of diverse sleep stages.

### Transmission Electron Microscopy

1 mm^3^ ipsilesional hippocampus tissues collected on day 28 were prepared as previously described (Wu et al., 2022). The samples were cut in ultrathin Sects. (70 nm) and stained with uranyl acetate and lead citrate. We observed the synapse ultrastructural changes using a transmission electron microscope (JEOL JEM-1400PLUS, Japan) and analyzed the vesicle number and postsynaptic density (PSD) length.

### Golgi-Cox Staining and Dendritic Spine Analysis

To study the morphological changes of the mouse hippocampus, the FD Rapid Golgi Stain Kit (FD NeuroTechnologies, PK401, USA) was applied to stain the neurons. Hippocampal samples collected on day 28 were prepared as per the manufacturer’s protocol. Brain slices were immersed in the impregnation solution and were stored in the dark for 2 weeks. The dendritic images were obtained using a laser-scanning confocal microscope (Zeiss LSM800, Germany), and analyzed using the Sholl Analysis plugin on the ImageJ software.

### Cell Culture, Transient Transfection, and Scratch Assay

Cultured from C57BL/6 mice brains on postnatal day 1 (P1), primary hippocampal neurons were dissociated and seeded at a density of 1,000,000 cells per well in a 6-well plate, following established procedures (Henderson et al., 2020). The culture medium consisted of Neurobasal medium (Gibco, 2,444,904, USA), supplemented with B27 (Gibco, 17,504,044, USA), 2 mM GlutaMax (Gibco, 35,050,061, USA), and 100 U/mL penicillin/streptomycin (Meilunbio, MA0110, China). Cells were incubated at 37 ℃ in a 5% CO2/95% air environment, with medium replacement every 3 days. Transient transfections were conducted using the Lipofectamine 3000 transfection kit (Thermofisher, USA) between days 5 and 12 in *vitro*. For scratch assays on day 13 in *vitro*, a sterile 10 μL pipette tip was used to create scratches along a 9 × 9 grid (4 mm intervals) on the 6-well plate surface, followed by medium replacement.

### Real-Time Quantitative PCR

The TRIzol reagent, following the manufacturer’s guidelines (Invitrogen, USA), was employed to extract total RNA from the samples. The cDNA Reverse Transcription Kit (Thermo Fisher, USA) was then used for cDNA synthesis. Subsequently, quantitative PCR was conducted using SYBR green (SYBR Green qPCR Master Mix, MedChemExpres, USA). The primers for FMOD were designed as 5′-CAACACCTTCAATTCCAGCA-3′ and 5′-ACCTGCAGCTGGGAGAAGT-3′, while those for GAPDH were chosen as 5′-GCACCACCAACTGCTTAGCACC-3′ and 5′-GTCTGAGTGTGGCAGGGACTC-3′.

### Western Blot

Proteins were extracted from both contralesional and ipsilesional hippocampus samples collected on day 28, as well as cells. The samples were loaded onto SDS-PAGE, followed by protein transfer to PVDF membranes (Millipore, USA). Incubation with primary antibodies (1:1000) was conducted overnight at 4 °C. Subsequently, bands were visualized using enhanced chemiluminescent substrates (Sigma-Aldrich, WBKLS0100, USA) after incubation with HRP-conjugated secondary antibodies at room temperature for 1 h. Internal control was maintained with β-actin (Zenbio, 200,068-8F10, China). Quantification and analysis of the gray value were performed using ImageJ software. Each experiment was replicated three times. The primary antibodies and reagents utilized included GFAP (ZEN BIO, 250,027, China), FMOD (GeneTex, GTX102783, USA), PSD95 (GeneTex, GTX22723, USA), SYP (GeneTex, GTX100865, USA), MAP2 (Proteintech, 17,490-1-ap, China), PI3K (Zenbio, 251,221, China), p-PI3K (Zenbio, 310,164, China), AKT (Zenbio, R23412, China), p-AKT (Zenbio, 310,021, China), mTOR (Zenbio, 380,411, China), p-mTOR (Zenbio, 381,557, China), and LY294002 (CAS: 154,447-36-6; Meilunbio).

### Immunofluorescence

After embedding in O.C.T. compound (Sakura Tissue-Tek, Japan), brain samples underwent rapid freezing in isopentane at − 80 °C for 5 min to ensure complete freezing. Subsequently, the cryostat was employed to prepare frozen brain sections. Cells, which were fixed with formaldehyde, and brain sections were subjected to an overnight incubation at 4 °C with the corresponding primary antibodies (1:100). Following this, they underwent incubation at room temperature with secondary antibodies conjugated with Fluor 488/594 (Invitrogen, 1:400, USA) for 1 h. Cell nuclei were identified using a culture medium containing DAPI (Solarbio, C0065, China). Results captured under a confocal microscope (Zeiss LSM800, Germany) were analyzed using ImageJ software. The primary antibodies used are as described previously.

### Statistical Analyses

Statistical analysis of the data from all experiments was conducted using GraphPad Prism version 9. Results are presented as mean ± SD. Homoscedasticity was assessed using Bartlett’s test. Comparisons between two independent groups were performed using a two-tailed t-test. Data sets involving more than two groups were analyzed using one-way or two-way ANOVA, followed by Tukey’s multiple comparisons test. Statistical significance is indicated as follows: *p < 0.05, **p < 0.01, ***p < 0.001, and ****p < 0.0001.

## Results

### FMOD is Significantly Downregulated After TBI in Patients and Mice

**Fig. 1:**
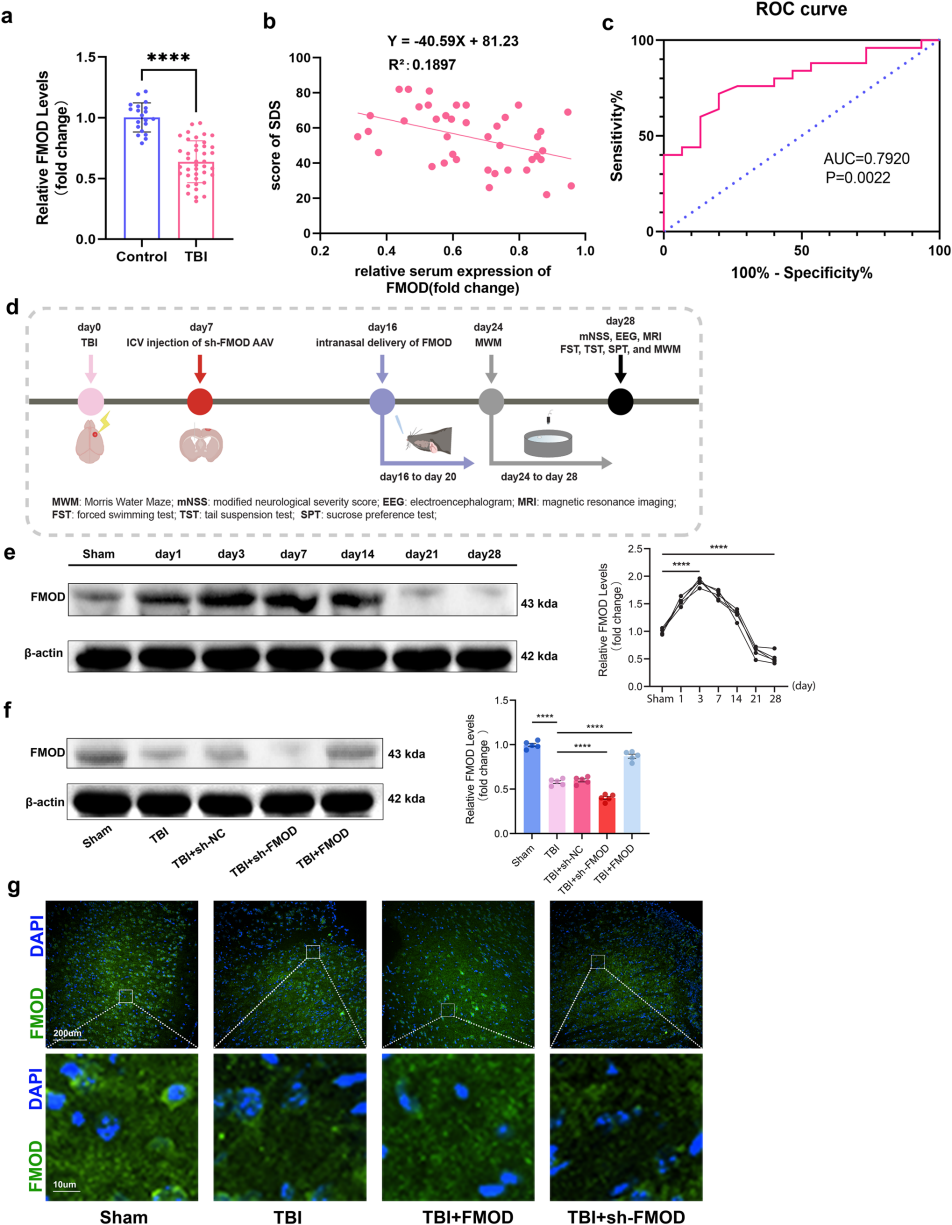
The expression levels of FMOD are diminished in patients and in mice after TBI. **a** The relative expression level of FMOD in the serum of TBI patients downregulated after TBI. n = 40 TBI patients, n = 20 volunteers as control, p < 0.0001, two-tailed t-test. **b**The depressive state was analyzed using the Self-Rating Depression Scale in patients 3 months after TBI. The scale score had a negative linear relationship with the expression of FMOD. *n* = 40 TBI patients, *n* = 20 volunteers as control, *p* = 0.0050. (**c**) ROC curve in the evaluation of the diagnostic value of serum FMOD for depression after TBI. Area under curve AUC = 0.7920; *p* = 0.0022. **d** Timeline of the experiment design. **e** Representative WB bands of FMOD showing the time course of FMOD expression after TBI, n = 5 mice per group. Sham group vs. day 3 group: *p* < 0.0001, Sham group vs. day28 group: *p* < 0.0001. **f** Representative WB bands of FMOD and on day 28 after FMOD delivery, n = 5 mice per group. Sham group vs.TBI group: *p* < 0.0001, TBI group vs. TBI + FMOD group: *p* < 0.0001, TBI group vs. TBI + sh-FMOD group: *p* < 0.0001. **g** Immunofluorescence results showed that FMOD was enriched in the ECM in mouse brain tissue. One-way ANOVA followed by Tukey’s test. All data were represented as mean ± SD

In this investigation, a significant reduction in FMOD levels within the serum of TBI patients, in comparison to healthy volunteers, was evident (*p* < 0.0001, Fig. 1a). Subsequent to mental state assessment using SDS, a noteworthy negative correlation emerged between SDS scores and FMOD expression levels (p < 0.05, Fig. 1b). Additionally, the ROC curve analysis suggested that FMOD levels could potentially function as a diagnostic marker for post-TBI depression in patients (p = 0.0022, Fig. 1c). The observed decrease in FMOD expression might be associated with TBI-induced depression.

The expression profile of FMOD in mice following TBI was explored. The experimental timeline is depicted in Fig. 1d. The time course study was performed to preliminarily evaluate the expression trajectory of FMOD following TBI. Compared with the Sham group, FMOD expression peaked on day 3 (*p* < 0.0001, Fig. 1e) and then gradually decreased to a significantly lower level on day 28 (*p* < 0.0001, Fig. 1e). To ensure that the intranasal delivery or knockdown of FMOD was successful, the FMOD expression levels on day 28 were measured and we found that TBI + FMOD group showed significantly higher FMOD levels than TBI group (*p* < 0.0001, Fig. 1f). The TBI + sh-FMOD group reached even lower level of FMOD compared with TBI group (*p* < 0.0001, Fig. 1f). Additionally, widespread distribution of FMOD in the ECM of the cerebral cortex tissue in normal mice was observed. However, 28 days post-TBI, a partial loss of its expression was detected (Fig. 1g). These findings indicated that FMOD may play a crucial regulatory role in TBI.

### FMOD Promotes Neurological Functional Recovery and Reduces Depression-Like Behaviors in the TBI Mouse Model

**Fig. 2:**
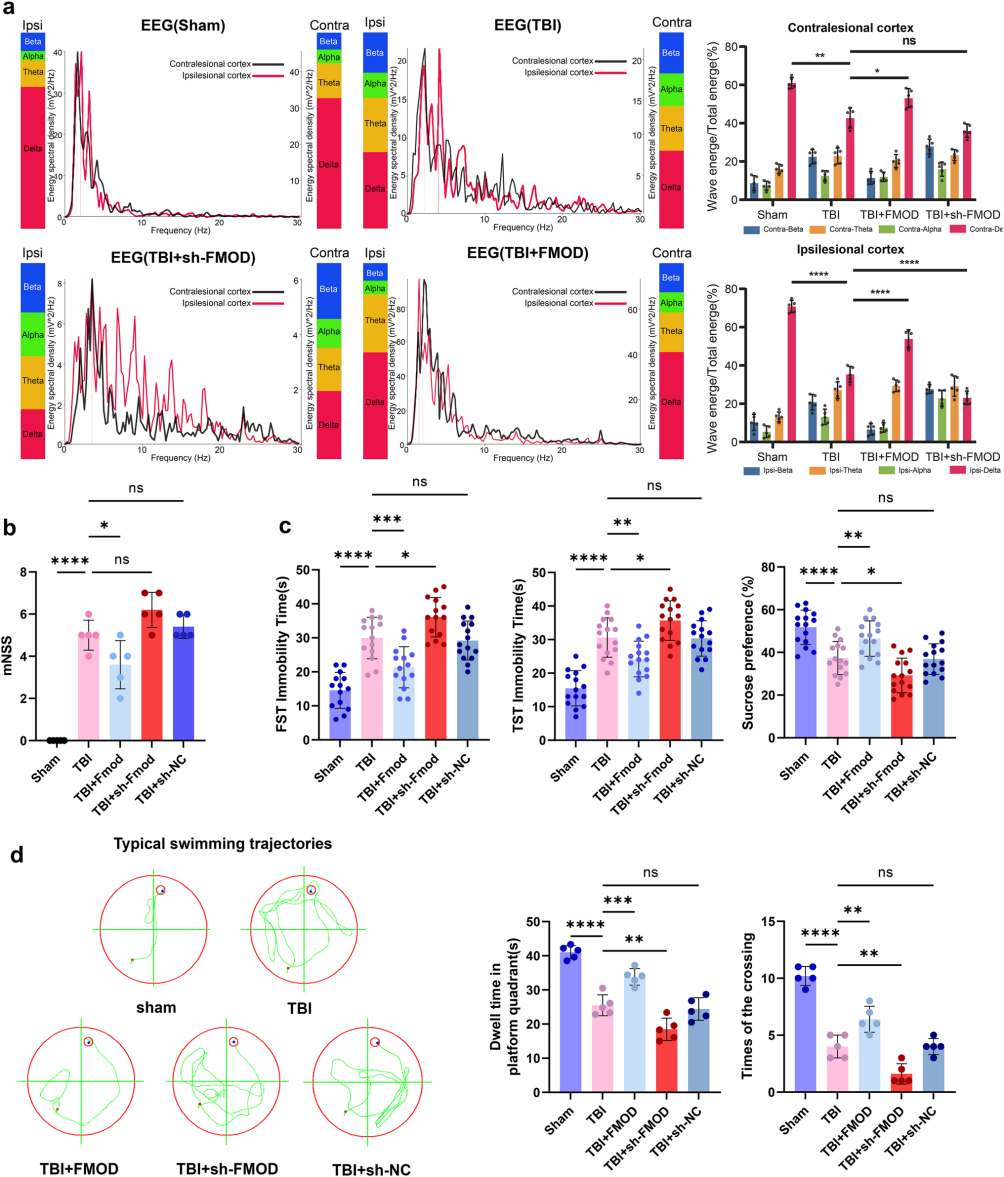
Overexpressing the FMOD alleviates sleep disorders and depression-like behaviors in mice after TBI. **a** Representative power spectral density estimated waveform corresponding to the frequency in the ipsilesional and contralesional cortex on day 28 after TBI, n = 5 mice per group. Contralesional: Sham group vs. TBI group: *p* < 0.01; TBI group vs. TBI + FMOD group: *p* < 0.05; TBI group vs. TBI + sh-FMOD group: ns, not significant; Ipsilesional: Sham group vs. TBI group:* p* < 0.0001; TBI group vs. TBI + FMOD group: *p* < 0.0001; TBI group vs. TBI + sh-FMOD group: *p* < 0.*0001*. **b** Modified Neurological Severity Scores (mNSS) were performed at 28d after TBI, n = 5 mice per group. Sham group vs. TBI group: *p* < 0.0001; TBI group vs. TBI + FMOD group: *p* < 0.05; TBI group vs. TBI + sh-FMOD group: ns, not significant. TBI group vs. TBI + sh-NC group: ns, not significant. **c** Depression-like behavior tests, including FST, TST, and SPF, n = 15 mice per group. The immobility time spent in the FST: Sham group vs. TBI group: *p* < 0.0001; TBI group vs. TBI + FMOD group: *p* < 0.001; TBI group vs. TBI + sh-FMOD group: *p* < 0.05; TBI group vs. TBI + sh-NC group: ns. The immobility time spent in the TST: Sham group vs. TBI group: *p* < 0.0001; TBI group vs. TBI + FMOD group: *p* < 0.01; TBI group vs. TBI + sh-FMOD group: *p* < 0.05; TBI group vs. TBI + sh-NC group: ns. The sucrose preference in the SPT: Sham group vs. TBI group: *p* < 0.0001; TBI group vs. TBI + FMOD group: *p* < 0.01; TBI group vs. TBI + sh-FMOD group: *p* < 0.05; TBI group vs. TBI + sh-NC group: ns. **d** Representative swimming trajectories of mice from each group on day 25; the big red circles indicate the pool edge; the small red circles indicate the platform locations. Time spent in the platform quadrant by each group of mice on day 28 when the platform was removed, n = 5 mice per group. Sham vs.TBI: *p* < 0.0001; TBI vs. TBI + FMOD: *p* < 0.001; TBI vs. sh-FMOD: *p* < 0.01; TBI vs. sh-NC: ns. Times of crossing through the platform quadrant of each group on day 28 when the platform was removed. (n = 5 mice per group). Sham vs.TBI: *p* < 0.0001; TBI vs. FMOD: *p* < 0.01; TBI vs. sh-FMOD: *p* < 0.01; TBI vs. sh-NC: ns. One-way ANOVA followed by Tukey’s test. All data were represented as mean ± SD

EEG analysis revealed a diminished spectral density of brain waves in the affected cerebral cortex of mice in the TBI group compared to the sham group. Specifically, the TBI + FMOD group exhibited a substantial augmentation in δ-wave energy within the affected cortex, in contrast to the TBI group. Meanwhile, the TBI + sh-FMOD group displayed a marked reduction in δ-wave energy (*p* < 0.0001, Fig. 2a**)**. Moreover, in comparison to the TBI group, the TBI + sh-FMOD group exhibited no significant difference in δ-wave energy in the contralateral lesion cortex (*p* > 0.05, Fig. 2a**)**. To summarize, EEG findings underscore the regulatory influence of FMOD on post-TBI sleep disturbances.

The mNSS outcomes indicated that FMOD led to a reduction in the mNSS score on day 28 after TBI (TBI + FMOD vs. TBI, *p* < 0.05, Fig. 2b), while no significant difference was observed between the TBI group and the TBI + sh-FMOD group (*p* > 0.05, Fig. 2b). This suggests that the overexpression of FMOD could enhance neurological recovery.

We assessed depression-like behavior in mice 28 days after TBI. In the final 4 min of both the FST and TST, mice in the TBI group exhibited varying degrees of increased floating or immobility compared to the Sham group (*p* < 0.0001, Fig. 2c). Administration of FMOD correspondingly reduced their duration of immobility (*p* < 0.01, *p* < 0.001, Fig. 2c). This suggested the emergence of depression-like behavior in mice post-TBI, with the overexpression of FMOD proving effective in ameliorating depressive states. Similar outcomes reflecting the influence of FMOD on depressive states were also observed in the SPT (*p* < 0.01, Fig. 2c).

The MWM is utilized as a behavioral paradigm to assess cognitive memory functions in mice. After a 5-day training phase, distinctive patterns in swimming trajectories emerged on day28 (Fig. 2d). Additionally, administering FMOD increased the time mice spent in the quadrant containing the platform (*p* < 0.01, *p* < 0.001, Fig. 2d) and enhanced their frequency of crossing the platform area (*p* < 0.01, Fig. 2d**)** after platform withdrawal on day 28. Conversely, the knockdown of FMOD reversed this effect (*p* < 0.01, Fig. 2d**)**. These findings suggested that FMOD may improve cognitive memory abilities in rodents, consistent with the previous report (Meng et al., 2016).

### Upregulating FMOD Improves Synaptic Plasticity by Enhancing the Expression of Synaptic Proteins

**Fig. 3:**
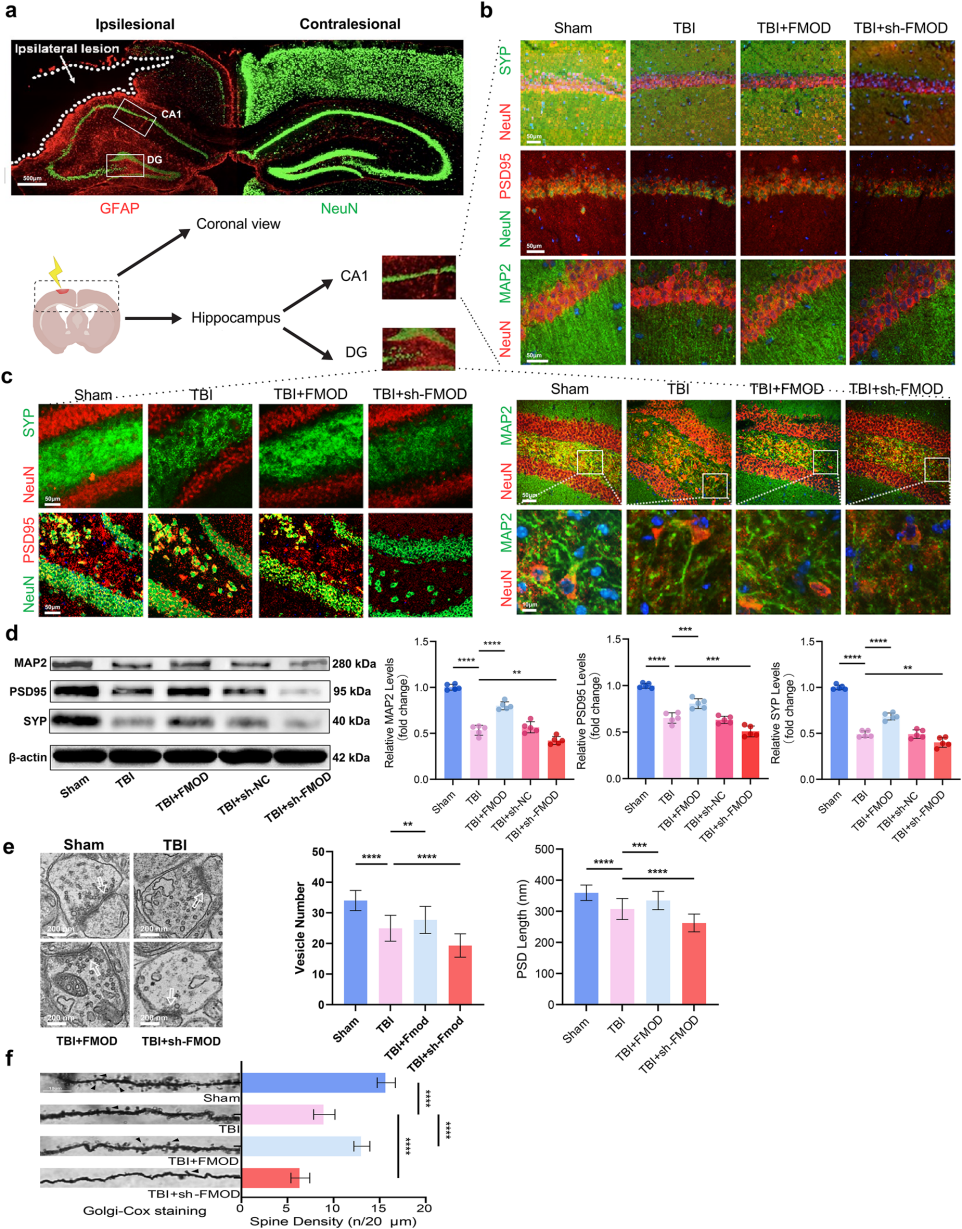
FMOD regulates synaptic plasticity in the hippocampus after TBI. **a** Representative IF images reveal the colocalization of NeuN and GFAP in the brain coronal section on day 28 after TBI. **b**–**c** Representative IF images show the co-localization of SYP, PSD95, and MAP2 in the hippocampal CA1 area and DG area of mice after upregulating or downregulating the FMOD, respectively. **d** Representative WB bands indicate the expression of MAP2, PSD95, and SYP in TBI mice after FMOD overexpression or knockdown, n = 5 mice per group. MAP2: Sham group vs. TBI group: *p* < 0.0001, TBI group vs. TBI + FMOD group: *p* < 0.0001, TBI group vs. TBI + sh-FMOD group: *p* < 0.01; PSD95: Sham group vs. TBI group: *p* < 0.0001, TBI group vs. TBI + FMOD group: *p* < 0.001, TBI + sh-NC group vs. TBI + sh-FMOD group: *p* < 0.001; SYP: Sham group vs. TBI group: *p* < 0.0001, TBI group vs. TBI + FMOD group: *p* < 0.0001, TBI group vs. TBI + sh-FMOD group: *p* < 0.01. **e** Electron microscope representative images of synapse ultrastructure of the CA1 neurons after FMOD overexpression or knockdown. White arrows point to synaptic structures, n = 40 cells per group. Vesicle number in each group: Sham group vs.TBI group: *p* < 0.0001, TBI group vs. TBI + FMOD group: *p* < 0.01, TBI group vs. TBI + sh-FMOD group: *p* < 0.0001. PSD length in each group: Sham group vs.TBI group: *p* < 0.0001, TBI group vs. TBI + FMOD group: *p* < 0.001, TBI group vs. TBI + sh-FMOD group: *p* < 0.0001. **f** Representative Golgi-Cox staining of CA1 neurons after FMOD overexpression or knockdown. Count the number of dendritic spines every 20 μm, n = 40 per group. Spine density: Sham group vs.TBI group: *p* < 0.0001, TBI group vs. TBI + FMOD group: *p* < 0.0001, TBI group vs. TBI + sh-FMOD group: *p* < 0.0001. One-way ANOVA followed by Tukey’s test. All data were represented as mean ± SD

TBI has a significant impact on synaptic structure and function, which is critical for synaptic plasticity and cognitive function, often resulting in synaptic loss (Jamjoom et al., 2021). Depression is commonly associated with TBI, accompanied by hippocampal neuronal atrophy, and diminished synaptic quantity and function (Kang et al., 2012). The regulation of synaptic formation and plasticity involves synaptic proteins SYP and PSD95. In our experiments, overexpressing FMOD was found to broaden the distribution of neurons and crucial synaptic proteins, PSD95 and SYP, after TBI. Furthermore, a distinct improvement in the continuity and integrity of the neuronal cytoskeleton protein MAP2 was evident with FMOD overexpression. These results implied that FMOD holds the potential to mitigate neuronal atrophy and synaptic loss caused by TBI (Fig. 3a–c). In addition, our study noted a broader distribution of GFAP on the injured side (Fig. 3a). The increased GFAP expression, a key constituent of glial scars, after TBI suggested a significant formation of glial scars, which could potentially hinder the establishment of intercellular synapses. The findings from the assessment of synaptic protein expression levels suggested that FMOD has the capacity to enhance the expression of MAP2, PSD95 and SYP (*p* < 0.001, *p* < 0.0001, Fig. 3d), while the downregulation of FMOD counteracts this effect (*p* < 0.01, *p* < 0.001, Fig. 3d). These results corroborated that FMOD can ameliorate neuronal atrophy and the reduction in synaptic numbers following TBI.

To further investigate synaptic plasticity, transmission electron microscopy was utilized to examine the ultrastructure of synapses. The results confirmed that overexpression of FMOD significantly increased both the number of synaptic vesicles and the length of PSDs after TBI (*p* < 0.001, Fig. 3e). Additionally, Golgi staining results showed that overexpression of FMOD stimulated the growth of new dendritic spines in neurons after TBI (*p* < 0.0001, Fig. 3f). Thus, FMOD showed potential in mitigating ultrastructural damage of synapses.

### FMOD Regulates Synaptic Proteins Through the PI3K/AKT/mTOR Signaling Pathway in *vivo *and in *vitro*

**Fig. 4:**
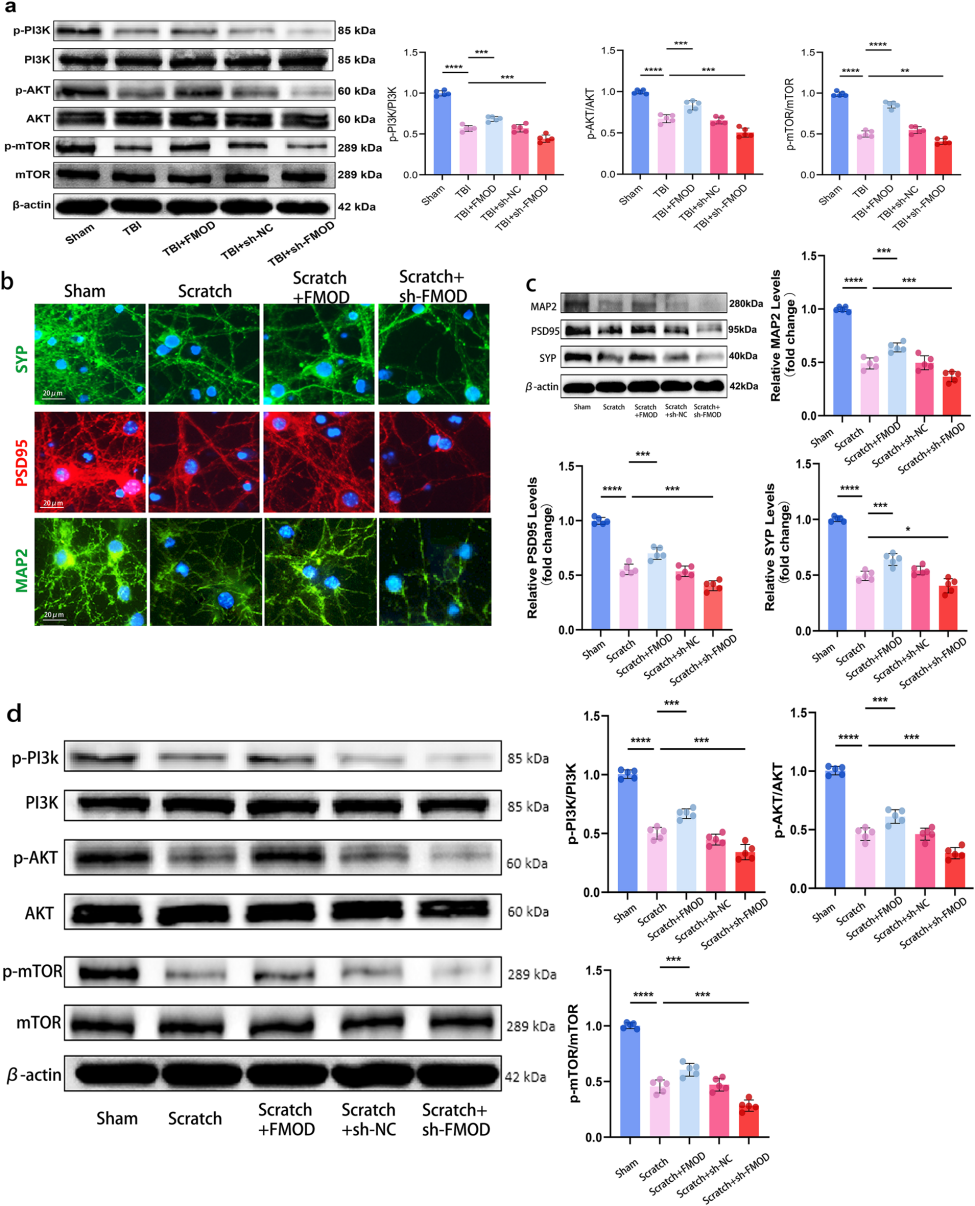
FMOD regulates synaptic proteins through the PI3K/AKT/mTOR signaling pathway in vivo and in *vitro*. **a** Representative WB bands display the expression of p-PI3K, PI3K, p-AKT, AKT, p-mTOR, and mTOR on day 28 in Sham, TBI, TBI + FMOD, TBI + sh-NC, and TBI + shFMOD groups, n = 5 mice per group. The phosphorylation ratio of PI3K: Sham group vs. TBI group: *p* < 0.0001, TBI group vs. TBI + FMOD group: *p* < 0.001, TBI group vs. TBI + sh-FMOD group: *p* < 0.001; The phosphorylation ratio of AKT: Sham group vs. TBI group: *p* < 0.0001, TBI group vs. TBI + FMOD group, *p* < 0.001, TBI group vs. TBI + sh-FMOD group, *p* < 0.001; The phosphorylation ratio of mTOR: Sham group vs. TBI group: *p* < 0.0001, TBI group vs. TBI + FMOD group: *p* < 0.0001, TBI group vs. TBI + sh-FMOD group: *p* < 0.01. **b** Representative IF images show the co-localization of SYP, PSD95, and MAP2 of primary hippocampal neuron cells in the group of Sham, Scratch, Scratch + FMOD, and Scratch + shFMOD. **c** Representative WB bands exhibit the expression of MAP2, PSD95, and SYP in Sham, Scratch, Scratch + FMOD, Scratch + sh-NC, and Scratch + shFMOD groups of the cells, n = 5. MAP2: Sham group vs. Scratch group: *p* < 0.0001, Scratch group vs. Scratch + FMOD group: *p* < 0.001, Scratch group vs. Scratch + sh-FMOD group: *p* < 0.001; PSD95: Sham group vs. Scratch group: *p* < 0.0001, Scratch group vs. Scratch + FMOD group: *p* < 0.001, Scratch group vs. Scratch + sh-FMOD group: *p* < 0.001; SYP: Sham group vs. Scratch group: *p* < 0.0001, Scratch group vs. Scratch + FMOD group: *p* < 0.001, Scratch group vs. Scratch + sh-FMOD group: *p* < 0.05. **d** Representative WB bands exhibit the expression of p-PI3K, PI3K, p-AKT, AKT, p-mTOR, and mTOR in Sham, Scratch, Scratch + FMOD, Scratch + sh-NC, and Scratch + shFMOD groups of the cells, n = 5 The phosphorylation ratio of each protein was calculated and analyzed. PI3K: Sham group vs. Scratch group: *p* < 0.0001, Scratch group vs. Scratch + FMOD group: *p* < 0.001, Scratch group vs. Scratch + sh-FMOD group:* p* < 0.001; AKT: Sham group vs. Scratch group: *p* < 0.0001, Scratch group vs. Scratch + FMOD group: *p* < 0.001, Scratch group vs. Scratch + sh-FMOD group: *p* < 0.001; mTOR: Sham group vs. Scratch group: *p* < 0.0001, Scratch group vs. Scratch + FMOD group: *p* < 0.001, Scratch group vs. Scratch + sh-FMOD group: *p* < 0.001. One-way ANOVA followed by Tukey’s test. All data were represented as mean ± SD

Evidence from studies has indicated the pivotal involvement of the AKT-mTOR signaling pathway in the activation of synaptic formation in rats (Vanderplow et al., 2021) and its significant role in the antidepressant process through this pathway (Li et al., 2010). Consequently, our investigation aimed to explore whether FMOD could augment synaptic plasticity by modulating this signaling pathway. Evaluation of protein expression in the mouse hippocampus revealed that FMOD activated the PI3K/AKT/mTOR signaling pathway, resulting in an increased phosphorylation rate (*p* < 0.001, Fig. 4a). Conversely, diminishing FMOD attenuated the expression of this pathway (*p* < 0.01, *p* < 0.001, Fig. 4a). These observations were further substantiated through in *vitro* experiments. In a simulated TBI utilizing a scratch injury model, the overexpression of FMOD led to densely packed cell synapses and an elevation in the expression of synaptic proteins (*p* < 0.05, *p* < 0.001, Fig. 4b, c). Additionally, FMOD induced the phosphorylation of the PI3K/AKT/mTOR signaling pathway in primary neuronal cells, while the knockdown of FMOD reversed this process (*p* < 0.01, *p* < 0.001, Fig. 4d).

**Fig. 5:**
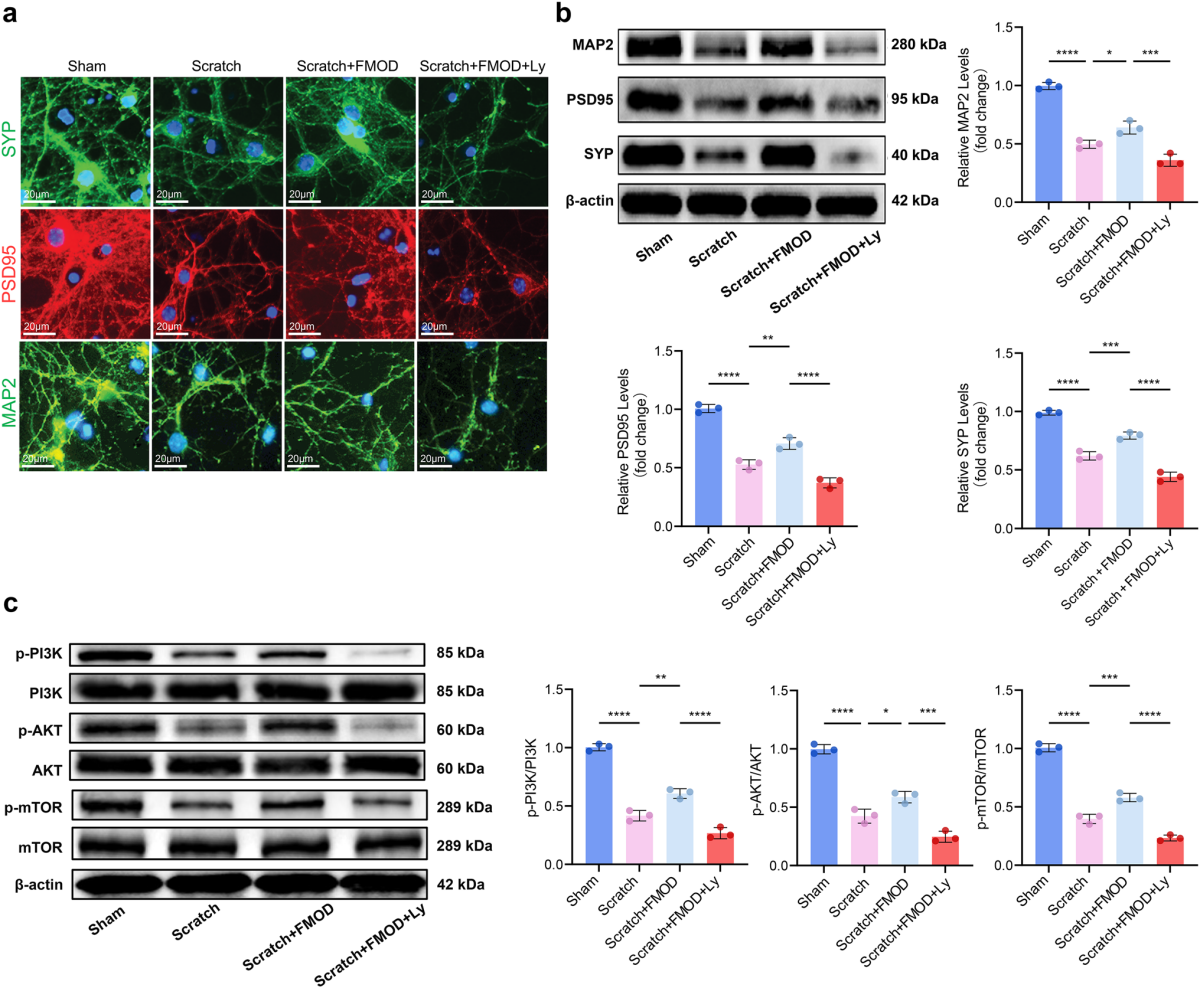
Inhibiting the PI3K/AKT/mTOR signaling pathway hinders the facilitative effect of FMOD. **a** Representative IF images show the co-localization of SYP, PSD95, and MAP2 of primary hippocampal neuron cells in the group of Sham, Scratch, Scratch + FMOD, and Scratch + FMOD + Ly. **b** Representative WB bands show the expression of MAP2, PSD95, and SYP in Sham, Scratch, Scratch + FMOD, Scratch + sh-NC, and Scratch + FMOD + Ly groups of the cells, n = 5. MAP2: Sham group vs. Scratch group: *p* < 0.0001, Scratch group vs. Scratch + FMOD group: *p* < 0.05, Scratch + FMOD group vs. Scratch + FMOD + LY294002 group:* p* < 0.001; PSD95: Sham group vs. Scratch group: *p* < 0.0001, Scratch group vs. Scratch + FMOD group: *p* < 0.01, Scratch + FMOD group vs. Scratch + FMOD + LY294002 group:* p* < 0.0001; SYP: Sham group vs. Scratch group: *p* < 0.0001, Scratch group vs. Scratch + FMOD group: *p* < 0.001, Scratch + FMOD group vs. Scratch + FMOD + LY294002 group: *p* < 0.0001. **c** Representative WB bands exhibit the expression of p-PI3K, PI3K, p-AKT, AKT, p-mTOR, and mTOR in Sham, Scratch, Scratch + FMOD, and Scratch + FMOD + Ly groups of the cells, n = 5. The phosphorylation ratio of each protein was calculated and analyzed. PI3K: Sham group vs. Scratch group: *p* < 0.0001, Scratch group vs. Scratch + FMOD group: *p* < 0.01, Scratch + FMOD group vs. Scratch + FMOD + LY294002 group: *p* < 0.0001; AKT: Sham group vs. Scratch group: *p* < 0.0001, Scratch group vs. Scratch + FMOD group: *p* < 0.05, Scratch + FMOD group vs. Scratch + FMOD + LY294002 group: *p* < 0.001; mTOR: Sham group vs. Scratch group: *p* < 0.0001, Scratch group vs. Scratch + FMOD group: *p* < 0.001, Scratch + FMOD group vs. Scratch + FMOD + LY294002 group: *p* < 0.0001. One-way ANOVA followed by Tukey’s test. All data were represented as mean ± SD

To ascertain the significance of the PI3K/AKT/mTOR pathway in the FMOD-mediated promotion of synaptic plasticity, we employed LY294002, a PI3K inhibitor, in primary neuronal cells to hinder the activation of the PI3K/AKT/mTOR signaling pathway. Immunofluorescence findings demonstrated that LY294002 effectively counteracted the stimulatory impact of FMOD on synaptic growth (Fig. 5a). Additionally, Western blot analysis confirmed that LY294002 impeded the FMOD-induced upregulation of SYP and PSD95 expression (*p* < 0.001, *p* < 0.0001, Fig. 5b). In addition, the pattern of protein expression of the PI3K/AKT/mTOR signaling pathway was in line with these observations. FMOD maintained the phosphorylation ratios of PI3K, AKT and mTOR in the Scratch + FMOD group (p < 0.05, p < 0.01, Fig. 5c). However, this protective effect was abolished by LY294002 administration (*p* < 0.001, *p* < 0.0001, Fig. 5c). In summary, these findings suggest that FMOD-induced synaptic plasticity post-TBI is contingent upon the activation of the PI3K/AKT/mTOR signaling pathway, culminating in an augmentation of synaptic protein levels.

## Discussion

This study elucidated the involvement of the ECM protein FMOD in the synaptic plasticity of depression after TBI for the first time. Initially, our findings suggest a correlation between depression and diminished levels of FMOD in the serum of TBI patients, it might be a biomarker of depression after TBI. Subsequent behavioral experiments in TBI mice elucidated the impact of FMOD on depressive states post-TBI. Furthermore, our observations in the hippocampus demonstrate that overexpression of FMOD mitigates TBI-induced neuronal atrophy, synaptic quantity reduction, and functional decline. Research into the potential mechanisms underlying FMOD's impact on synaptic function in depressive behavior unveiled that FMOD, acting through the PI3K/AKT/mTOR axis, alleviated TBI-induced synaptic functional impairments, thereby ameliorating depressive states.

Nowadays, studies have broadly reported numerous peripheral biomarkers in depression, like the heterotrimeric G protein, Gsalpha (Targum et al., 2022), serum uric acid (X. Meng et al., 2020), and the miRNA in serum (Lopez et al., 2018). Nevertheless, only a few research indicated the serum biomarkers in TBI-related depression. Several studies found that the expression level of tau protein in the serum might be a meaningful biomarker to predict depressive symptoms after TBI (Lange et al., 2023, 2024; Pattinson et al., 2020). Another study illustrated that the Brain-Derived Neurotrophic Factor (BDNF) was preliminarily associated with depression following TBI. In this study, the SDS served as a tool for preliminary screening and assessing the severity of depressive states after TBI. Then, we drew the blood of TBI patients at admission and assessed the expression level of FMOD. The consequences showed that the decrease in FMOD levels in TBI patients might be linked to the onset of depression post-TBI. Furthermore, we observed the dynamic expression pattern of FMOD in TBI mice, which showed a swift increase in FMOD expression, followed by a gradual decline to significantly lower levels. The important role of FMOD in neurological disorders has been gradually recognized in recent years. FMOD has been reported to orchestrate gene expression in the central position following TBI through transcriptomic analysis in rat TBI model (Meng et al., 2017). Our outcomes verified again the role of FMOD in TBI and further suggested that it might be a novel serum biomarker to predict post-TBI depression.

In recent years, it has been demonstrated that FMOD plays a crucial role in regulating neurological function and metabolic diseases (Meng et al., 2016). Intriguingly, depression was associated with diminished volumes of critical brain structures involved in cognitive regulation, specifically the prefrontal cortex and hippocampus (Price & Duman, 2020), marked by neuronal atrophy and synaptic inhibition in the medial prefrontal cortex and hippocampus (Duman & Aghajanian, 2012; Duman et al., 2016). However, scarcely any research discovered the correlation between FMOD and depression after TBI. In our study, the EGG results of mice illustrated that the impairment of δ wave activity could be mitigated by FMOD, additionally, the MWM test demonstrated that FMOD improved the cognitive memory function of mice, which all showed that FMOD might concurrently influence depressive states and cognitive function after TBI. Subsequently, the localization and expression of PSD95, SYP, and MAP2 of the CA1 and DG area in the hippocampus were detected, and then the synapse ultrastructure and dendritic spines of neurons in the CA1 area were observed, these outcomes implicated the FMOD could elevate the expression of synapse-related proteins and the density of dendritic spines, attenuates the ultrastructure damages of synapse post-TBI. FMOD is a crucial structural component of the ECM, and the function of the ECM in the regulation of neurite outgrowth, neural plasticity, and functionality was increasingly recognized (Dityatev et al., 2010). These findings support the previous study above and demonstrate that FMOD may have an effect on depressive symptoms and cognitive function through modulation of the prefrontal cortex and hippocampus after TBI.

In addition, as a proteoglycan present in the ECM, FMOD is a potent mediator of growth factor-related signaling pathways (Zheng et al., 2017). For example, FMOD could modulate TGF-β1 activities via transient phosphorylation of SMAD2, enhanced phosphorylation of SMAD3, and suppressed AP-1-mediated non-canonical TGF-β1 signaling transduction, ultimately significantly reducing scar formation in animal cutaneous wounds (Zheng et al., 2017). While the PI3K/AKT/mTOR signaling pathway was identified as involved in the biological process both in TBI and depression (Feng et al., 2022; Y. Wu et al., 2023). Additionally, it has been demonstrated that the knockdown of FMOD leads to a decrease in the phosphorylation of the PI3K/AKT/mTOR signaling pathway, resulting in the inhibition of both proliferation and migration of retinal pigment epithelial cells (Hu et al., 2018). This finding is consistent with our results. However, further research is required to investigate how FMOD regulates the pathophysiological changes associated with depression related to traumatic brain injury.

In conclusion, our results indicated that FMOD is a novel therapeutic target for depression after TBI treatment by activating the PI3K/AKT/mTOR signaling pathway, thereby improving synaptic plasticity, and ameliorating depression-like behaviors.

## Supplementary Information

Below is the link to the electronic supplementary material.

Supplementary file1 (DOCX 25 KB)

Supplementary file2 (DOCX 15 KB)

## Data Availability

The datasets generated and analyzed during the current study are available from the corresponding author on reasonable request.
