# Supplementary material for: FMOD Alleviates Depression-Like Behaviors by Targeting the PI3K/AKT/mTOR Signaling After Traumatic Brain Injury
Source: Neuromolecular Med. 2024 Jun 12;26(1):24. doi: 10.1007/s12017-024-08793-2 (PMC11169026; doi:10.1007/s12017-024-08793-2)
Supplement: Supplementary file 1 — Supplementary file1 (DOCX 25 KB) [file 12017_2024_8793_MOESM1_ESM.docx]

**Supplementary Table 1.** Clinical data from control and TBI cases.

| NO. Group Age Gender BMI GCS |
| --- |
| \| 1 \| control \| 43 \| F \| 22.6 \| 15 \| \| --- \| --- \| --- \| --- \| --- \| --- \| \| 2 \| control \| 29 \| F \| 25.2 \| 15 \| \| 3 \| control \| 37 \| M \| 23.1 \| 15 \| \| 4 \| control \| 40 \| M \| 19.3 \| 15 \| \| 5 \| control \| 29 \| F \| 25.2 \| 15 \| \| 6 \| control \| 46 \| M \| 23.9 \| 15 \| \| 7 \| control \| 28 \| F \| 24.5 \| 15 \| \| 8 \| control \| 33 \| M \| 22.2 \| 15 \| \| 9 \| control \| 35 \| F \| 23.6 \| 15 \| \| 10 \| control \| 28 \| F \| 24.8 \| 15 \| \| 11 \| control \| 36 \| M \| 21.1 \| 15 \| \| 12 \| control \| 19 \| F \| 24.2 \| 15 \| \| 13 \| control \| 33 \| F \| 26.3 \| 15 \| \| 14 \| control \| 30 \| F \| 25.1 \| 15 \| \| 15 \| control \| 37 \| M \| 23.9 \| 15 \| \| 16 \| control \| 20 \| F \| 22.8 \| 15 \| \| 17 \| control \| 45 \| M \| 18.3 \| 15 \| \| 18 \| control \| 37 \| M \| 25.1 \| 15 \| \| 19 \| control \| 42 \| M \| 23.7 \| 15 \| \| 20 \| control \| 38 \| M \| 19.2 \| 15 \| \| 21 \| TBI \| 50 \| F \| 21.2 \| 10 \| \| 22 \| TBI \| 33 \| F \| 18.9 \| 11 \| \| 23 \| TBI \| 57 \| F \| 21.0 \| 11 \| \| 24 \| TBI \| 48 \| M \| 23.5 \| 9 \| \| 25 \| TBI \| 41 \| M \| 23.4 \| 13 \| \| 26 \| TBI \| 33 \| M \| 25.0 \| 11 \| \| 27 \| TBI \| 28 \| M \| 24.6 \| 9 \| \| 28 \| TBI \| 44 \| M \| 20.8 \| 8 \| \| 29 \| TBI \| 36 \| F \| 23.0 \| 12 \| \| 30 \| TBI \| 28 \| M \| 22.1 \| 10 \| \| 31 \| TBI \| 42 \| F \| 25.6 \| 8 \| \| 32 \| TBI \| 39 \| M \| 23.8 \| 11 \| \| 33 \| TBI \| 40 \| F \| 21.4 \| 10 \| \| 34 \| TBI \| 33 \| M \| 22.6 \| 9 \| \| 35 \| TBI \| 19 \| F \| 22.9 \| 11 \| \| 36 \| TBI \| 36 \| M \| 22.0 \| 10 \| \| 37 \| TBI \| 22 \| F \| 23.7 \| 12 \| \| 38 \| TBI \| 33 \| M \| 20.9 \| 13 \| \| 39 \| TBI \| 28 \| F \| 20.4 \| 8 \| \| 40 \| TBI \| 43 \| F \| 23.3 \| 11 \| \| 41 \| TBI \| 36 \| M \| 23.6 \| 11 \| \| 42 \| TBI \| 54 \| F \| 24.7 \| 8 \| \| 43 \| TBI \| 43 \| F \| 20.5 \| 13 \| \| 44 \| TBI \| 49 \| M \| 22.6 \| 8 \| \| 45 \| TBI \| 37 \| F \| 24.3 \| 10 \| \| 46 \| TBI \| 48 \| M \| 20.9 \| 9 \| \| 47 \| TBI \| 34 \| M \| 22.4 \| 12 \| \| 48 \| TBI \| 47 \| F \| 23.4 \| 11 \| \| 49 \| TBI \| 29 \| F \| 19.0 \| 13 \| \| 50 \| TBI \| 30 \| F \| 23.0 \| 8 \| \| 51 \| TBI \| 49 \| F \| 19.1 \| 10 \| \| 52 \| TBI \| 32 \| F \| 17.2 \| 8 \| \| 53 \| TBI \| 47 \| M \| 22.1 \| 10 \| \| 54 \| TBI \| 53 \| M \| 22.9 \| 9 \| \| 55 \| TBI \| 34 \| M \| 19.9 \| 13 \| \| 56 \| TBI \| 31 \| F \| 23.2 \| 10 \| \| 57 \| TBI \| 49 \| M \| 24.5 \| 11 \| \| 58 \| TBI \| 32 \| M \| 20.1 \| 10 \| \| 59 \| TBI \| 38 \| M \| 22.4 \| 12 \| \| 60 \| TBI \| 39 \| M \| 25.8 \| 9 \| |
