# Supplementary material for: FMOD Alleviates Depression-Like Behaviors by Targeting the PI3K/AKT/mTOR Signaling After Traumatic Brain Injury
Source: Neuromolecular Med. 2024 Jun 12;26(1):24. doi: 10.1007/s12017-024-08793-2 (PMC11169026; doi:10.1007/s12017-024-08793-2)
Supplement: Supplementary file 2 — Supplementary file2 (DOCX 15 KB) [file 12017_2024_8793_MOESM2_ESM.docx]

**Table S2. Exact sequence of sh-RNA used to knock down FMOD expression.**

| **Name** | **Sequence** |
| --- | --- |
| sh-FMOD | 5’-CCGGTGTCTCACAACAGTCTCACTACTCGAGTAGTGAGACTGTTGTGAGACATTTTTT-3’ |
| sh-NC | 5’-CCGGGGTTCTCCGAACGTGTCACGTCTCGAGACGTGACACGTTCGGAGAACCTTTTTG-3’ |
